# Isolation, Characterisation, and Lipase Production of a Cold-Adapted Bacterial Strain *Pseudomonas* sp. LSK25 Isolated from Signy Island, Antarctica

**DOI:** 10.3390/molecules24040715

**Published:** 2019-02-16

**Authors:** Leelatulasi Salwoom, Raja Noor Zaliha Raja Abd Rahman, Abu Bakar Salleh, Fairolniza Mohd. Shariff, Peter Convey, David Pearce, Mohd Shukuri Mohamad Ali

**Affiliations:** 1 Enzyme and Microbial Technology Research Center, Faculty of Biotechnology and Biomolecular Sciences, Universiti Putra Malaysia, Serdang Selangor 43400, Malaysia; lt.salwoom@gmail.com (L.S.); rnzaliha@upm.edu.my (R.N.Z.R.A.R.); abubakar@upm.edu.my (A.B.S.); fairolniza@upm.edu.my (F.M.S); 2 National Antarctic Research Centre (NARC) B303, Block B, Level 3, IPS Building, University of Malaya, Kuala Lumpur 50603, Malaysia; 3 Department of Microbiology, Faculty of Biotechnology and Biomolecular Sciences, Universiti Putra Malaysia, Serdang 43400, Selangor, Malaysia; 4 British Antarctic Survey, NERC, High Cross, Madingley Road, Cambridge CB3 OET, UK; pcon@bas.ac.uk (P.C.); david.pearce@northumbria.ac.uk (D.P.); 5 Department of Applied Sciences, Faculty of Health and Life Sciences, University of Northumbria at Newcastle, Newcastle-Upon-Tyne NE1 8ST, UK; 6 Department of Biochemistry, Faculty of Biotechnology and Biomolecular Science, Universiti Putra Malaysia, Serdang Selangor 43400, Malaysia

**Keywords:** Antarctica, cold adapted lipase, *Pseudomonas* sp. LSK25, lipase production

## Abstract

In recent years, studies on psychrophilic lipases have been an emerging area of research in the field of enzymology. This study focuses on bacterial strains isolated from anthropogenically-influenced soil samples collected around Signy Island Research Station (South Orkney Islands, maritime Antarctic). Limited information on lipase activities from bacteria isolated from Signy station is currently available. The presence of lipase genes was determined using real time quantification PCR (qPCR) in samples obtained from three different locations on Signy Island. Twenty strains from the location with highest lipase gene detection were screened for lipolytic activities at a temperature of 4 °C, and from this one strain was selected for further examination based on the highest enzymatic activities obtained. Analysis of 16S rRNA sequence data of this strain showed the highest level of sequence similarity (98%) to a *Pseudomonas* sp. strain also isolated from Antarctica. In order to increase lipase production of this psychrophilic strain, optimisation of different parameters of physical and nutritional factors were investigated. Optimal production was obtained at 10 °C and pH 7.0, at 150 rev/min shaking rate over 36 h incubation.

## 1. Introduction

Extreme environments including those of Antarctica have been successfully colonised by numerous microorganisms and the biodiversity of these microorganisms is becoming increasingly well documented. These microorganisms, which are typically psychrophilic or psychrotolerant, have developed various adaptations enabling them to survive the harsh effects of such environments [1,2]. Enzymes are increasingly reported as an essential component in their adaptation, receiving attention owing to their relevance for both basic and applied research. Increasing effort is being focused in the search for cold-adapted enzymes with potential application in biotechnological industries [3,4].

True psychrophiles are able to grow at temperatures below 10 °C. They are also known as extremophiles due to their ability to adapt to low temperature and other extreme environmental stresses [5]. Since their entire cellular processes can take place in a cold environment it is crucial that all components of the cell, including metabolism and protein synthesis, are well adapted to function at low temperatures. Pivotal features include the maintenance of functional membranes, the evolution of cold-adapted enzymes, and the inclusion of a range of structural features which endow a high level of flexibility in protein structure while maintaining the active site, high catalytic efficiency at low temperatures, high degree of thermolability, and a lower energy of activation [6,7,8].

The properties of cold-adapted enzymes such as lipases (CLPs) offer a wide range of potentially beneficial biotechnological applications in fields as wide as the detergent, textile and food industries, and in the bioremediation of polluted soils and wastewater treatment [9,10]. CLPs break down fats and belong to the wider class of enzymes that catalyse hydrolysis reactions (hydrolases). CLPs have evolved specific structural features which provide thermal flexibility around the active site and high specific activity at low temperatures [11]. Such characteristics have paved the way for their use in various industrial applications, such as leather processing, medical and pharmaceutical preparations, fine chemical synthesis, detergent additives, food processing, and environmental bioremediation [12,13,14,15]. To date, many studies have focused on the production of lipase enzymes from thermophiles [16,17,18], and little attention has been paid to the potential for the production of cold-adapted lipases sourced from the microbiota of extremely cold environments.

This study set out to isolate, identify and quantify the occurrence of cold-adapted lipases in bacterial strains obtained from anthropogenically-influenced areas close to the British Antarctic Survey’s Signy Island research station (South Orkney Islands, maritime Antarctic), and to further analyse physical and nutritional factors that may be used to enhance lipase production by the strains obtained.

## 2. Results

### 2.1. Screening for Cold Adapted Lipase

**Figure 1:**
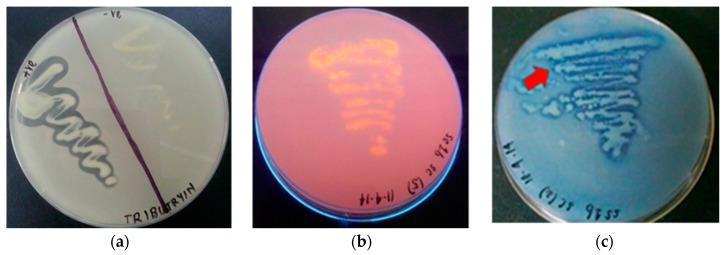
(**a**) Lipolytic activity clearing zone on tributyrin agar; (**b**) lipolytic activity on Rhodamine B, with substrate hydrolysis leading to the formation of an orange fluorescent halo upon exposure to UV radiation; and (**c**) lipolytic activity confirmation due to change in (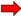) colour of dye indicators on triolein agar.

Microscopic analysis revealed that the majority of the strains isolated were rod-shaped, Gram negative bacteria. A total of 63 bacterial isolates were obtained from the Signy Island soil samples, and were screened for extracellular lipase production on tributyrin agar plates. Only 20 isolates produced a zone of clearance (Figure 1a) surrounding each colony after 3 d incubation at 4 °C, indicating the hydrolysis of tributyrin by the lipolytic activity of the isolates. However, tributyrin is not only susceptible to hydrolysis by lipases, but also by esterases. Of the 20 isolates tested, only 11 showed positive evidence for lipase production on both triolein and Rhodamine B (Figure 1b,c) agar plates.

**Figure 2:**
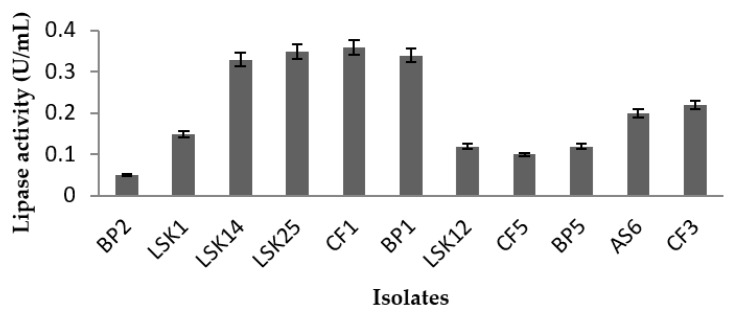
Lipase activity of 11 isolates that tested positive in qualitative analysis. Lipase activities are represented by mean value ± standard deviation (n = 3).

All 11 isolates were then subjected to the protocol described by Kwon and Rhee (1986) [19]. As the objective was to screen and obtain cold-adapted lipases, all screening was carried out at 4 °C. Amongst the 11 isolates, strains LSK25, LSK14, CF1, and BP1 recorded the highest lipase activity (Figure 2), this being in the range of 0.35 to 0.4 U/mL, and these four strains were selected for the next stage of study.

### 2.2. Lipase Gene Quantification via qPCR

The top four lipase producing strains were subjected to quantification of the cold-adapted lipase gene activity by qPCR, based on the standard curve produced using the empty plasmid pGEM^®^-T. The quantification clearly showed the isolate LSK25 had the highest number of lipase gene copies, 2.45 × 10^14^ copies/µL (Table 1). Hence this isolate was selected for further study.

### 2.3. Strain Characterisation

**Figure 3:**
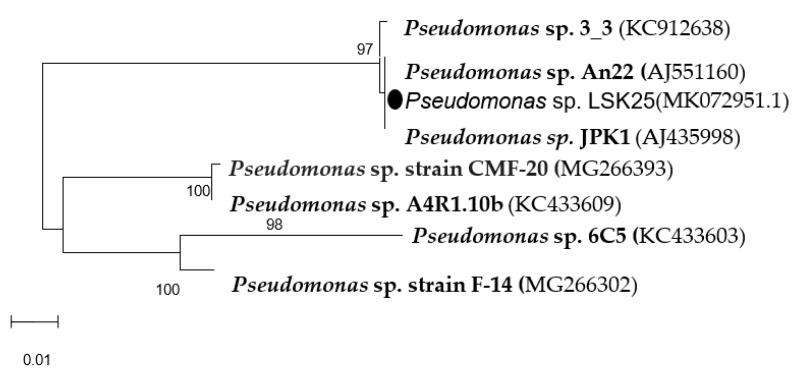
Unrooted Neighbour Joining tree illustrating the relationship of strain *Pseudomonas* sp. LSK25 (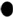) with other *Pseudomonas* sp. strains obtained from the polar regions.

Isolate LSK25 showed gram staining morphological characteristics of a Gram negative aerobic rod, and was catalase and oxidase positive. The isolate demonstrated no haemolysis (gamma), and can therefore be concluded to be non pathogenic. Microbact analysis revealed that the isolate was able to metabolise various carbohydrates, including pentoses, methylpentose, and hexoses. The isolate failed to hydrolyse citrate and urea, suggesting it is not a member of the Enterobacteriaceae, which also have similar Gram staining morphology. 16S rDNA sequence analysis revealed similarity to the bacterial genus Pseudomonas, with 98% similarity to *Pseudomonas* sp. An22 and *Pseudomonas* sp. JPK1, isolated from different parts of the Antarctic (Figure 3). Partial 16S rRNA gene sequence of *Pseudomonas* sp. LSK25 was deposited in the GenBank database under the accession number MK072951.1

### 2.4. Effect of Physical Parameters on Lipase Production

**Figure 4:**
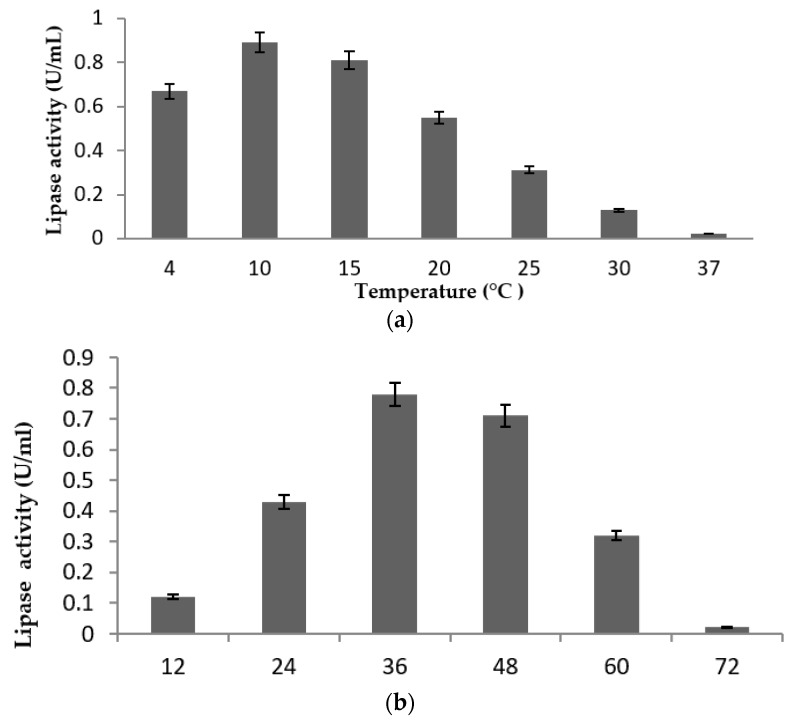

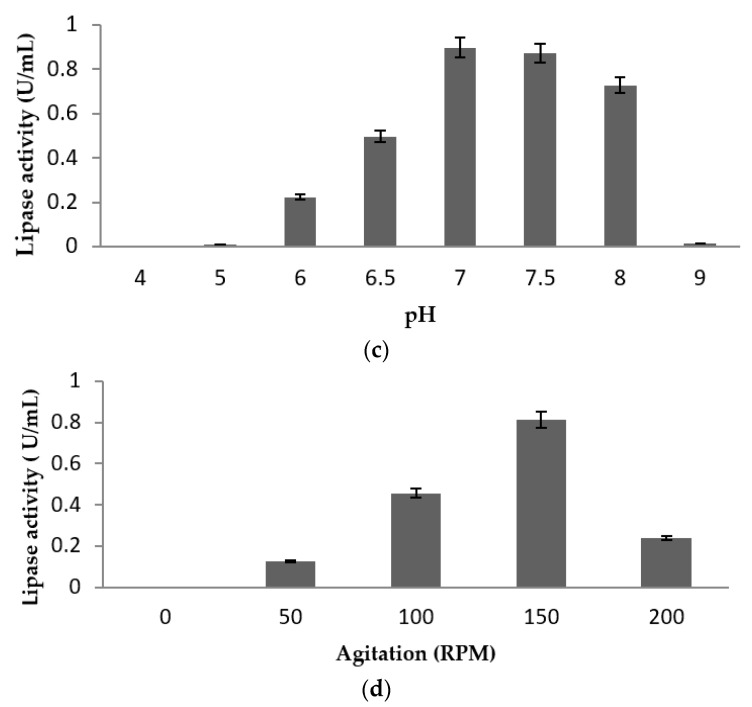
Lipase activities represented by mean value ± standard deviation (n = 3). (**a**) Effect of temperature on lipase activity of strain of LSK25 measured at 4–37 °C. (**b**) Effect of incubation time on activity of lipase in strain LSK25 over 12 h intervals. (**c**) Effect of pH of production medium on lipase activity in strain LSK25 over a pH range of 4 to 9. (**d**) Effect of agitation of production medium on lipase activity in strain LSK25, over the range 0 to 200 rpm.

Cultures of the strain were incubated at different temperature ranging from 4 to 37 °C to investigate the optimum temperature for lipase production. Highest lipase activity was observed at the low temperature (Figure 4a) of 10 °C. Lipolytic activity started to be detected after 12 h incubation, reaching maximum at 36 h. A steep decline in the activity was apparent after 48 h incubation (Figure 4b). The optimum pH range for lipase activity is between 7.0 and 8.0 (Figure 4c). Activity was drastically reduced at acidic (4.0 and 5.0) and alkaline pH (9.0). The highest lipase activity was achieved at 150 rpm (Figure 4d) over 36 h at 10 °C in production medium at pH 7.0. Significant differences were apparent across the ranges tested for each physical parameter (ANOVA, all *p* < 0.05) and all post hoc pairwise comparisons were significant.

### 2.5. Effect of Nutritional Parameters on Lipase Production

**Figure 5:**
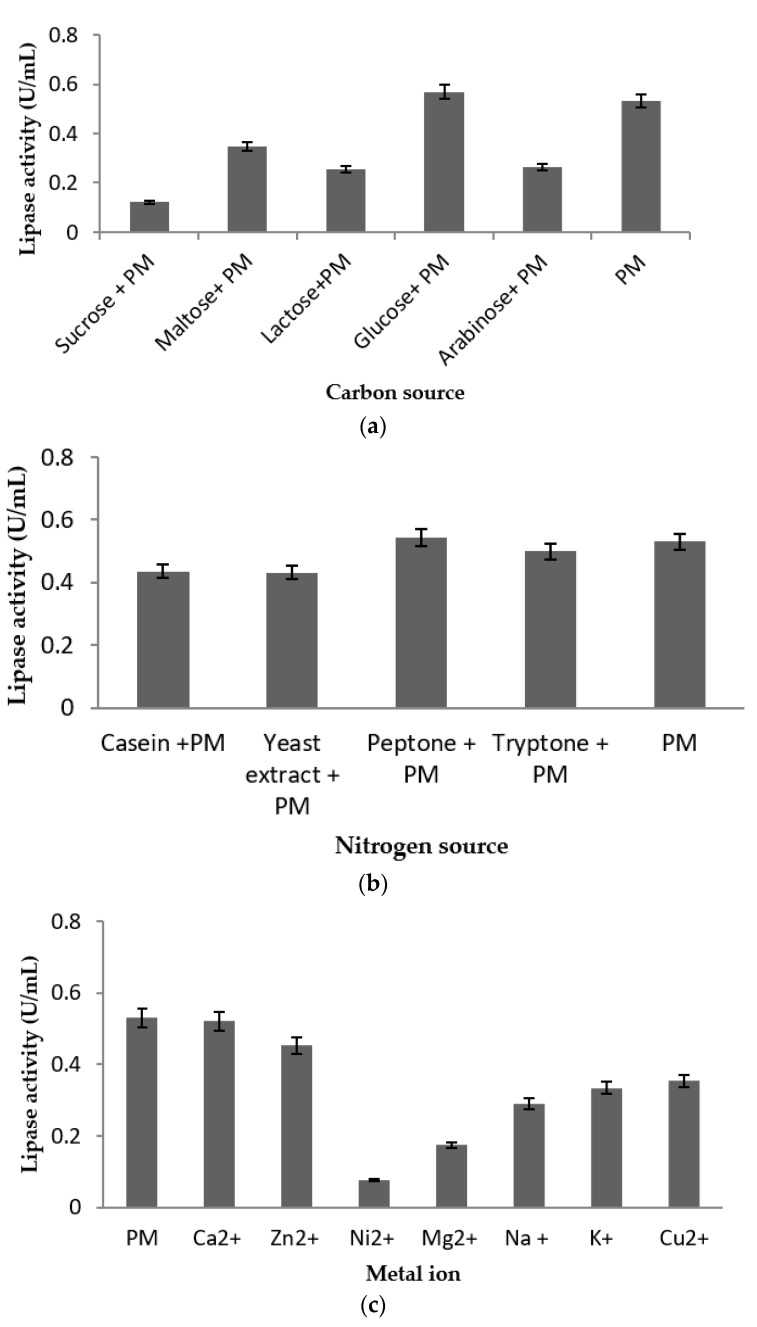

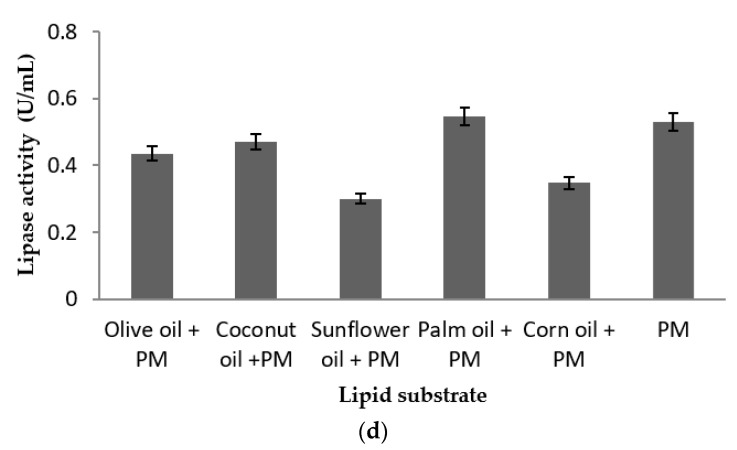
Lipase activities represented by mean value ± standard deviation (n = 3). (**a**) Influence of carbon source on lipase activity of strain LSK25. Carbon sources were added to the media to a final concentration of 1% (*w*/*v*). (**b**) Influence of nitrogen source on lipase activity of strain LSK25. The nitrogen sources were added to a final concentration of 0.8% (*w*/*v*). (**c**) Influence of various metal ions on lipase activity of strain LSK25. The metal ions in nitrate form were added to a final concentration of 1 mM. (**d**) Influence of various natural triglycerides on lipase activity of strain LSK25. Olive oil in production medium was substituted with the other substrates to a concentration of 1% (*v*/*v*) (PM = Production medium).

The carbon sources tested in this study strongly influenced enzyme production. Only the addition of glucose slightly increased the lipase activity obtained, by 12% in comparison to production medium. One-way ANOVA revealed significant differences. The addition of sucrose, maltose, lactose, and arabinose significantly reduced the lipase production generally by more than 50% (Figure 5a). Figure 5b illustrates the effects of different organic nitrogen sources added separately into the production medium. The data obtained illustrate that an approximately 5% increase in lipase production occurred when 0.8% peptone was added to the production medium. Again ANOVA and post hoc comparisons revealed these differences to be significant. Addition of the remaining nitrogen sources resulted in slight decreases in lipase activity in comparison to the production medium alone.

Among the metal ions tested in this study (Figure 5c), only the addition of Ca^2+^ resulted in an increase in lipase production, of 2%, while the remaining metal ions suppressed lipase production. The transition metals, especially Ni^2+^, led to reductions in activity of up to 88%, with somewhat lesser reductions associated with Mg^2+^, Na^+^, K^+^, and Cu^2+^. Different effects on lipase production were observed when provided with different natural triglycerides as substrate (Figure 5d). Palm oil led to an increase of 10% in production, while sunflower oil led to the greatest suppression of up to 50%. The differences were significant for both of these parameters (ANOVA, all *p* < 0.05) and post hoc pairwise comparisons.

## 3. Discussion

Psychrophiles are defined as microbes that are able to thrive at low temperature (below 10 °C), and are currently underutilised in biotechnological applications globally. These microorganisms possess cold-adapted enzymes which are important in the maintenance of metabolic functions in extreme climates [20,21,22]. The low temperature lipolytic activity of isolates obtained in the current study from Signy Island confirms their ability to hydrolyse the long chain fatty acids contained in tributryin, olive oil, and triolein.

It is widely accepted that abiotic factors such as pH and temperature can have a strong influence on enzymatic processes, as well as cell membrane permeability [23]. Lipases can metabolise well in a wide range of pH and temperature conditions, and bacterial lipases are most commonly effective in an alkaline medium [24]. Maximum lipase production in the current study was obtained between pH 7 and 8 (Figure 4c), consistent with the review of Gupta et al. (2004) [24]. The preference of strain LSK25 for neutral to slightly alkaline conditions mirrors reports from other cold-adapted lipase producers, including *Pseudomonas antarctica* sp. nov., *P.. meridiana* sp. nov. [25], *Pseudomonas* sp. strain KB700 [26], *Acinetobacter* sp. strain no. 6 [13], *Aeromonas* sp. LPB 4 [27], and *P. aeruginosa* [28]. As shown in Figure 4d, very low lipase production was detected at acidic (pH 4.0 and 5.0) and alkaline conditions (pH 9.0). Rapid changes in pH may alter the production of bacterial metabolites [29,30], including changes in lipase production [18]. The isolate examined here has potential as a source of enzymes for use in specific industrial processes that take place in conditions similar to the optima identified. The slightly alkalophilic nature of the strain is appropriate for improving the performance and stability of biological treatment systems designed for the bioremediation of water (contaminated with chlorinated solvents, hydrocarbons, nitrates, and other biologically degradable compounds), detergent formulations, sewage treatment, and leather processing [30].

A pivotal factor in microbial enzyme cultivation is the optimum temperature that stimulates catalytic site optimum saturation, while higher temperature eventually denatures the enzyme [31]. Psychrophiles tend to have good enzymatic production rate at low temperature, hence the production of cold-adapted lipase enzyme is fundamentally temperature dependent and thermolabile [26]. Maximum lipase production in the current study was obtained after 36 h incubation at 10 °C, with the rate subsequently declining, most likely due to the depletion of nutrients available to the cells in the production medium [32]. Lipolytic activity also decreased with increasing temperature whereas optimum lipase production and activity temperatures were similar (Figure 4a). The crude enzyme exhibited maximum lipolytic activity at 15 °C, slightly higher than the 10 °C optimum production temperature, using olive oil as substrate. Lower lipase production at higher temperatures than 15 °C might be due to limitation in the amount of total dissolved oxygen in the medium, and the solubility of oxygen further decreases with rising temperature [18,33]. Similar data have been reported from other microbes of cold-adapted lipases, producers including *Curtobacterium* sp. [30] and the yeast *Candida* sp. KKU-PH2-15 [34].

Medium agitation is a key factor that enhances bacterial growth and the production of cold-adapted lipase by improving the rate of oxygen transfer and increasing the dispersal of oil micelles enabling them to come into contact with the microbial cell [31,35,36]. The rate of culture agitation had a significant effect on lipase production in strain LSK25. An agitation rate of 150 rpm led to the highest lipase activity, consistent with other reports on *Pseudomonas* spp. [37,38,39] (Figure 4d). Higher agitation rates lead to enhanced oxygenation of the culture medium [18]. The sharp decline noted at 200 rpm is generally associated with enzyme denaturation and also the accumulation of hydrogen peroxide, a damaging by-product [40,41].

Plausible biotechnological applications of low temperature, high lipase activity strains include as detergent additives or in the processing of volatile substances due to their ability to reduce processing temperature and thereby bring down energy costs [15]. The abiotic parameters of strain LSK25 documented here, with optimum lipase production at 10 °C and near-neutral pH, are similar to those of cold-adapted lipase enzymes currently used in industrial applications such as cheese ripening [42], food storage [43], water treatment, cold water detergents [44], and cosmetics [45]. Carbon and nitrogen sources are pivotal for the maintenance of cellular functions and growth [46], including enzyme production. In industrial applications, appropriate sources can also significantly reduce production costs. Lipase production in relation to different carbon and nitrogen sources has been studied in fermentation processes [47,48], and carbon sources derived from saccharides are known to significantly influence lipase production [49]. The influence of different carbon sources on lipase production varied considerably in the current study (Figure 5a). However, of the carbon sources tested, only glucose led to a small but significant increase in production in comparison to the production medium alone. The remaining carbon sources led to lower lipase production, consistent with the results of the Microbact analysis. The one-way ANOVA test proved there were significant differences between the production medium alone and with the addition of glucose. Sabat et al. (2012) similarly reported that some microorganisms showed higher lipase activities when grown in media containing glucose [50], as did the fungal strain *Candida* sp. KKU-PH2-15 [34]. Rathi et al. (2001) also reported that addition of 1% glucose led to an increase in enzyme production in *Burkholderia cepacia* [51]. Glucose is considered as an economically beneficial carbon source which can enhance the yield and increase the production of cold-active lipases [32].

The organic nitrogen source, peptone, increased the lipase production of LSK25 by as much as 19% in comparison to the production medium (Figure 5b). Other studies have also shown that the addition of peptone into production media increases the production of lipase enzymes; including *Aneurinibacillus thermoaerophilus* strain HZ [16], *Bacillus coagulans* BTS-3 [52], and the cold-adapted lipase strain *Pseudomonas* sp. strain B11-1 [9]. Addition of tryptone, yeast extract, and casein resulted in slightly lower lipase production, while beef extract reduced production. Such data indicate either the inability of this strain to hydrolyse these nitrogen sources, or that the products released were toxic to the strain [16,53].

The data obtained in this study indicate that strain LSK25 requires a simple production medium, with the addition of the calcium alone enhancing lipase production. The negative impact on lipase production especially of nickel and other transition metal ions mirrors data presented by Joseph et al. (2006) [44]. In the current study palm oil, an economical and widely available natural triglyceride generated lipase activity similar to that of the production media alone, followed by olive oil and then coconut oil. The ability of strain LSK25 to hydrolyse long chain fatty acids mirrors that reported in *Pseudomonas* sp. S5 [23].

## 4. Materials and Methods

### 4.1. Study Sites

Signy Island (60°43′ S 45°36′ W) is a maritime Antarctic island within the South Orkney Island archipelago. The annual soil temperature is approximately −2 °C and annual precipitation is approximately 400 mm/year [54,55].

### 4.2. Soil Sampling

Surface soil samples were collected from three sites on Signy Island during the austral summer season 2006/2007 (Table 2). At each sampling location, six replicate soil samples of approximately 50 g were collected from the top 5 cm of the soil profile using sterile falcon tubes. Samples were kept at 4 °C immediately after collection and frozen on station at the earliest opportunity (−20 °C).

### 4.3. Bacterial Culture

The soil samples were cultured into normal strength Luria-Bertani (LB) broth. The samples were first sieved to remove large particles such as stones and plant debris. Approximately 1 g of each sample was inoculated into separate aliquots of 10 mL LB broth and grown at 4 °C in a 150 rpm refrigerated orbital shaker incubator (LM-510RD, YihDer Tech. Co., Taiwan) for 72 h. Growth density was measured using a spectrophotometer (SmartSpec^TM^ PLUS BioRad, Hercules, CA, USA). Once an initial optical density at 600 nm of 0.5 was achieved, the bacterial cultures were individually inoculated onto LB agar normal strength plates for single colony streaking. All incubation from this point onwards took place at 4 °C. Single colonies of each culture were then inoculated into LB broth and cultured for 72 h, after which 80% glycerol stocks were made and stored at −80 °C. Single colonies were viewed under the light microscope to determine bacterial morphology.

### 4.4. Screening of Cold-Adapted Lipase

#### 4.4.1. Qualitative Analyses

The isolates were enriched in nutrient broth at 4 °C under shaking conditions (150 rpm) at an initial pH 7.0. The enriched cultures were screened qualitatively on tributyrin agar plates, consisting of tributyrin at 1.0%, *v*/*v*. Lipolytic activity were confirmed through the formation of clear zones around the colony. Positive isolates were then further tested for lipase production on Rhodamine B (0.001%, *w*/*v*), and Victoria Blue (0.01%, *w*/*v*) agar plates containing tributyrin, triolen, or olive oil as substrate [56]. Lipolysis was observed directly at 4 °C incubation over 48 to 72 h by changes in the appearance of the substrate, such as formation of a clear zone and change in colour of the indicator dye used [57]. Rahman et al. (2009) demonstrated a linear logarithmic relationship between the diameter of the diffusion spot on lipase selection plates and enzyme concentration, confirming this methodology is appropriate for comparing the effectiveness of lipase production [16]. An analogous method of detection (tributyrin and Rhodamine B agar plates) has also been used previously to study the lipolytic activity of *Pseudomonas* sp. strain B11-1 [9] and *Pseudomonas* sp. AMS8 [58].

#### 4.4.2. Quantitative Analyses

Quantification of lipase activity was conducted by extracting the free fatty acid (FFA) released with isooctane and colouring with copper reagent (Kwon & Rhee, 1986) [19]. A standard curve of oleic acid was generated to permit quantification of the lipase activity. A single colony of each lipase producer was picked off the nutrient agar plate and inoculated into 10 mL of nutrient broth. After 72 h incubation at 4 °C with shaking, 1%, *v*/*v* of each culture was inoculated into 50 mL of production medium (peptone (0.75%, *w*/*v*), olive oil (1.0%, *v*/*v*), yeast extract (0.3%, *w*/*v*), 0.5% glucose, 0.05% CaCl_2_.2H_2_O, 1% FeCl_3_.6H_2_O) [40,59]. The isolates were then incubated for an additional 72 h at 4 °C while shaking at a constant rate. Lipase activity was assessed by a simple and rapid colorimetric method modified from Kwon and Rhee (1986), using olive oil as substrate [19]. The reaction mixture, consisting of 1.0 mL of crude enzyme, 2.5 mL olive oil emulsion (1:1 ratio of olive oil and phosphate buffer [K_2_HPO_4_ (50 mM), KH_2_PO_4_ (50 mM)] at pH 7.0) and 0.02 mL of 20 mM CaCl_2_.2H_2_O, was incubated in a water bath shaker at 4 °C for 30 min with an agitation rate of 200 rpm. The reaction was then terminated by the addition of 1.0 mL of 6 N HCl. The FFAs were subsequently extracted by the addition of 5.0 mL isooctane and vigorous mixing using a vortex mixer for 30 s. The upper isooctane layer (4 mL) containing the FFAs were transferred to a test tube containing 1 mL of copper reagent and vortexed again. The reagent was prepared by adjusting the solution of 5% (*w*/*v*) copper (II) acetate-1-hydrate to pH 6.1 with pyridine. The amount of FFAs dissolved in the isooctane layer was measured at 715 nm using a spectrophotometer (SmartSpec^TM^ PLUS BioRad, Hercules, CA, USA). One unit of lipase activity was defined as the release one micromole of free fatty acid in one min.

#### 4.4.3. Characterisation of the Strains

Strains were characterised using biochemical and molecular techniques. Morphology and motility were determined by phase contrast microscopy and Gram staining, and biochemical tests were carried out using commercial Microbact^TM^ biochemical analysis kits (Thermoscientific, USA). Microbact^TM^ analysis is a standardised test of microsubstrates designed to mimic conventional biochemical substrates used for the identification of common aerobic and facultative anaerobic Gram negative bacilli (MGNB) whereby pH changes and substrate utilisations are used for microorganism identification [60]. The genomic DNA of the isolate which produced the highest lipase activity was then extracted using the DNeasy Tissue Kit (Qiagen, Germany), following the manufacturer’s instructions. This was then used as the template to perform Polymerase Chain Reaction (PCR) amplification of 16S rDNA for identification. The 16S rDNA was amplified using two universal primers: 16S-F (5′-AGA GTT TGA TCC TGG CTC AG-3′) and 16S-R (5′-ACG GCT ACC TTG TTA CGA CTT-3′). The amplified product was electrophoresed on 1% agarose gel (*w*/*v*) with a 1 kb DNA marker (Thermo Scientific, USA). The PCR product was visualised under UV light and purified using the QIAquick gel extraction kit (Qiagen, Germany). The 16S rDNA was sequenced and an apparent homology search was performed using the GenBank database (NCBI).

### 4.5. Absolute Quantification (qPCR)

The Applied Biosystems, USA 7500 Fast Real-Time PCR system (Applied Biosystems, USA) was used for qPCR amplification and detection. qPCR was prepared in triplicates of 20 or 25 μL reaction mixture in MicroAmp optical 96-well reaction plates and sealed with optical adhesive covers (Applied Biosystems, USA). Three replicates of a control sample without DNA template were also included in the runs**.** The primer pair F/R-AMS8 [61] was first evaluated using a SYBR Green protocol. These primers were used as they yielded the approximate fragment size suitable for qPCR analysis, in the range 200 to 300 bp, and targeted the conserved region of cold-adapted lipases. To optimise the amount of primers and template DNA in the reactions, cDNA extraction was used. The tested concentrations ranged from 700 to 200 nM for primers and from 10 ng to 0.1 pg for template DNA. The optimised SYBR Green protocol was carried out in a final volume of 25 μL, containing 1.0 ng of template DNA, 12.5 μL of 2× SYBR^®^ Premix Ex Taq^TM^ (BioLine), 0.5 μL of 50× ROX^TM^ Reference Dye (BioLine), and 400 nM of both primer pairs [60]. The following thermal cycling conditions were used by the SYBR Green method: a single step of 10 min at 95 °C, 40 cycles of 95 °C for 15 s, and 60 °C for 1 min. After the final PCR cycle, melting curve analysis of the PCR products was performed by heating to 60–95 °C and continuous measurement of the fluorescence to verify the PCR product. Threshold cycle (Ct) value, corresponding to the PCR cycle number at which fluorescence was detected above threshold, was calculated by the 7500 Fast System SDS software (Applied Biosystems, USA). Standards were made from 10-fold dilutions of linearised plasmids of pGEM^®^-T Easy Vector Systems (Promega, USA) containing the gene fragment of interest that was cloned from amplified pure culture DNA (lipase gene, R^2^ = 0.999).

### 4.6. Effect of Physical and Nutritional Factors on Bacterial Growth and Lipase Production

To investigate the influence of physical nutritional factors on the growth and extracellular lipase production of strain lipLSK25, an overnight bacterial culture (OD_600_ = 0.5) was inoculated into production medium and incubated for 36 h. Physical parameters (pH, growth temperature, time of incubation, and agitation) and nutritional parameters (carbon, nitrogen, metal ion, and lipid substrates) were examined for their influences on lipase production and bacterial growth. Lipase assay was performed using the modified method of Kwon and Rhee (1986), as described above [19].

#### 4.6.1. Effect of Temperature, Incubation Time pH and Agitation

The ability of strain lipLSK25 to grow and produce lipase enzymes was measured at temperatures of 4, 10, 15, 20, 25, 30, and 37 °C over 36 h incubation with agitation at 150 rpm. The effect of pH was studied by adjusting the media (with 1.0 M NaOH or 6N HCl) to pH values from 4 to 9. The effect of agitation was examined using agitation rates of 0, 50, 100, 150, and 200 rpm. The production of lipase was assayed over time at intervals of 12 h. All the cultures were incubated in the designated orbital shaker (LM-510RD, YihDer Tech. Co., Taiwan), cultured in 1L Duran Scott bottles (Sigma Aldrich, St. Louis, MO, USA).

#### 4.6.2. Effect of Nutritional Factors on Bacterial Growth and Lipase Production

To investigate the influence of nutritional factors on extracellular lipase activity by strain lipLSK25, an overnight bacterial inoculum (OD_600_ = 0.5) was inoculated into production medium as described in Section 4.4.2. The initial pH was adjusted to 7.0 and incubation took place at 10 °C with shaking at 150 rpm for 36 h. To test the effect of different carbon sources the following were added to the production medium at 1% (*w*/*v*): sucrose, maltose, arabinose, lactose, or glucose. All carbon sources were separately sterilised through a 0.22-µm membrane filter. The organic nitrogen sources used were casein, yeast extract, tryptone, peptone, added to a final concentration of 0.8% (*w*/*v*). The effects of metal ions were determined by addition of K^+^, Na^+^, Mg^2+^, Ca^2+^, Zn^2+^, Cu^2+^, Fe^2+^, or Ni^2+^ in nitrate form to a final concentration of 1 mM. To investigate the effects of substrate-related compounds (natural oil at 1%, *v*/*v*), olive oil was substituted with the following: sunflower oil, palm oil, coconut oil and or corn oil. All the cultures were incubated in the designated orbital shaker (LM-510RD, YihDer Tech. Co., Taiwan), cultured in 1L Duran Scott bottles (Sigma Aldrich, Germany).

### 4.7. Statistical Analyses

Each experiment was repeated at three times. One-way ANOVA was used to compare within treatment group using SPSS Statistics v21 (IBM, Amund City, NY, USA), and where significant means were then compared by Tukey’s test at the 0.05 level of confidence.

## 5. Conclusions

A cold adapted lipase producer *Pseudomonas* sp. LSK25 was successfully isolated from Signy station, Antarctica. The data obtained demonstrated that LSK25 lipase production was dependent on nutritional and physical parameters. The ability of this isolate to exhibit high lipase activity at low temperature combined with a cost effective simple production medium, offers attractive potential commercial applications.

## Figures and Tables

**Table 1:** Lipase gene copy quantification using qPCR.

Isolates	Lipase Gene Copy Numbers (n = 3)	s.d. (±)
LSK14	1.02 × 10^10^	0.16
LSK25	2.45 × 10^14^	0.11
CF1	6.35 × 10^8^	0.12
BP1	3.04 × 10^10^	0.11

**Table 2:** The study sites sampled on Signy Island.

Sampling Sites	Description	Location
Area surrounding Signy Station (LSK)	Around the station living quarters, with high human activity, old whaling station	60°42′56”S 45°36′22”E
Bernsten Point (BP)	Site of previous Signy station buildings, historical oil spillages	60°40′16”S 45°34′42”E
Cemetery flats (CF)	Site of historical whaling industry activity, including oil spills and other pollution	60°41′16”S 45°38′42”E
